# Group discussion: Challenges and key elements to be addressed when transferring GP

**DOI:** 10.1093/eurpub/ckac129.510

**Published:** 2022-10-25

**Authors:** J Txarramendieta, J Roca, M Zahorka, K Strand Kudajewski, R Papa, Z Dolecek

**Affiliations:** 1 Kronikgune Institute for Health Services Research, Barakaldo, Spain; 2 August Pi i Sunyer Biomedical Research Institute, Barcelona, Spain; 3 Optimedis AG, Hamburg, Germany; 4 Health Innovation Centre of Southern Denmark, Odense, Denmark; 5 Regional Health Agency Marche Region, Ancona, Italy; 6 University Hospital Olomouc, Olomouc, Czechia

## Abstract

In this group discussion, all speakers will participate in a group discussion. The moderator will launch questions to speakers, and attendees will be encouraged to make contributions to the group discussion.

